# Socioeconomic Status Affecting Inequity of Healthcare Utilisation in Malaysia

**DOI:** 10.21315/mjms2019.26.4.9

**Published:** 2019-08-29

**Authors:** Nurul Salwana Abu Bakar, Adilius Manual, Jabrullah Ab Hamid

**Affiliations:** Institute for Health Systems Research, Ministry of Health Malaysia, Shah Alam, Selangor, Malaysia

**Keywords:** equity, healthcare utilisation, socioeconomic status, inpatient, outpatient

## Abstract

**Background:**

Equity is one of the important aspects of universal health coverage. Variation in socioeconomic status (SES) has been proved to contribute discrepancies in the use of healthcare services. This study aimed to assess equity for inpatient, outpatient and dental care utilisation by household SES over time.

**Methods:**

This study used five series of National Health and Morbidity Survey data from 1986 to 2015. Healthcare utilisation for inpatient, outpatient and dental care were analysed. SES was grouped based on household expenditure variables accounting for total number of adults and children in the household using consumption per adult equivalents approach. The determination of healthcare utilisation across the SES segments was measured using concentration index.

**Results:**

The overall distribution of inpatient utilisation tended towards the pro-poor, although only data from 1996 (*P*-value = 0.017) and 2006 (*P*-value = 0.021) were statistically significant (*P* < 0.05). Out-patient care showed changing trends from initially being pro-rich in 1986 (*P* < 0.05), then gradually switching to pro-poor in 2015 (*P* < 0.05). Dental care utilisation was significantly pro-rich throughout the survey period (*P* < 0.05). Public providers mostly showed significantly pro-poor trends for both in- and out-patient care (*P* < 0.05). Private providers, meanwhile, constantly showed a significantly pro-rich (*P* < 0.05) trend of utilisation.

**Conclusion:**

Total health utilisation was close to being equal across SES throughout the years. However, this overall effect exhibited inequities as the effect of pro-rich utilisation in the private sector negated the pro-poor utilisation in the public sector. Strategies to improve equity should be consistent by increasing accessibility to the private sectors, which has been primarily dominated by the richest population.

## Introduction

Equity in healthcare is defined as equal access for equal need, equal utilisation for equal need, and equal quality of care for all (1); it is the basis of the universal health coverage (UHC) concept. UHC can be achieved by ensuring all people can obtain health services they require without experiencing financial hardship (2), which is an aspiration of all countries. Progress towards UHC in the Southeast Asia region has been excellent in both preventive and curative care services (3). Malaysia has emphasised to ensure access to quality healthcare services across all communities (4), and has claimed to have achieved UHC in terms of financial protection and small out-of-pocket payments, as well as equitable usage distribution of public services (5).

Malaysia’s healthcare system exhibits a dichotomous model of healthcare services with a public-private mix. Public healthcare providers in Malaysia impose a nominal charge (or exemption) on users, focusing to cater the poor population and those in rural areas to ensure equitable access for those in need, whereas private sector providers are funded primarily by out-of-pocket payment and private insurance, and are usually located in urban areas (6). Access to and utilisation of private facilities is usually limited to richer society owing to affordability constraints (7).

Socioeconomic status (SES) has been widely used in studies and touted as a good predictor of health (8), because it is correlated with education, income, and occupation (or a composite of these three), and ties to affordability of goods consumption (9). Regarding inequitable spending and health services utilisation, a local study has reported that the richer population (higher SES) has a higher utilisation of private sector providers, whereas lower SES populations utilise public healthcare more (10). Thus, the patterns of public and private utilisation are influenced by SES, and equity in healthcare utilisation is directly linked with SES (11). Indeed, disparities in healthcare utilisation across the SES segments can be observed.

Presently, rising healthcare costs is a phenomenon faced by most developing countries and has put more constraints on the public sector (12). Theoretically, healthcare utilisation shifts from private to public sector when users can no longer afford private sector services. Reducing poor–rich inequity in healthcare has recently become one of the most important priorities of national governments and international organisations, and promoting equity is one of the main challenges of the health sector. The establishment of the value of these differences and quantification of the size of inequity are prerequisites for achieving this goal. Therefore, it is important to determine the current extent of inequality that possibly exists in Malaysia and how it has changed in the last decades. This study aimed to assess equity in healthcare utilisation by household SES over time using five series of survey data of the National Health Morbidity Survey (NHMS), indicated by concentration index (CI).

## Methods

### Data Source

Related information on population characteristics and healthcare utilisation was sourced from NHMS, a nationally representative household survey that has been conducted periodically since 1986. This survey has been an important platform in providing population data for Malaysian health monitoring. The design of the questionnaire has undergone slight changes over time, implying that some estimates may not be fully comparable. For example, the 1986 NHMS did not include questions on dental care utilisation and the 2006 NHMS did not capture inpatient days. Nonetheless, NHMS data are the most comprehensive and best sources available at the population level. Details of the survey methodology are described elsewhere (13).

### Outcome and Associated Variables

In the present study, utilisation was defined as the self-reported number of visits to any health facility (for outpatient and dental care) and number of days being hospitalised for inpatient care (with the exception of 2006, when number of visits was used), including separate use of public and private providers. Recall period was two weeks for outpatient care, and one year for inpatient and dental.

The SES of individual respondents was measured using the consumption per adult equivalents (AE) approach: individuals are ranked based on household expenditure, accounting for the total household members (adults and children). This approach has been widely used by the Organisation for Economic Co-operation and Development (OECD) countries (14), and its detailed information has been described elsewhere (15). SES was categorised into five quintiles, where 20% of the poorest and those in fifth quintile are the richest 20%. Missing data on expenditure variable were imputed relative to education level and employment status.

### Statistical Analysis

Relative inequalities of the utilisation distribution were measured using CI across the SES segments. Ranging from −1 to +1, CI summarised the direction and degree on the concentration area of utilisation. Negative values implied that the utilisation was concentrated among the poor (pro-poor) and vice-versa for positive values (pro-rich). A value of 0 indicated equal distribution across SES. Details on the concept and formula have been described elsewhere (16). All analyses were conducted using STATA version 13 (Stata Corp, Texas, USA), taking into account the sample weight and study design, with statistical significance set at *P* < 0.05.

## Results

Ideally, utilisation proportion should be distributed equally across the five SES segments, giving 20% value for each quintile. Any proportion exceeding 20% can be considered over-utilisation (and vice-versa). For the total inpatient care (Table 1), the first, second, and fourth quintiles (Q1, Q2, and Q4, respectively) generally had slightly more utilisation. For outpatient care, the values were stable at around 20% across the period and SES segments. For dental care, the difference was more distinguishable: the richer populations were utilising it more, especially the Q5, from year 2011 onwards.

When total utilisation was separated to public (Table 2) and private (Table 3) use, a different trend was observed. The two poorest quintiles generally had more utilisation of the public sector. Meanwhile, the richest population, particularly Q5, noticeably had lower public sector utilisation. This trend applied for both inpatient and outpatient care. For the private sector, clearly only the two richest quintiles utilised them, especially Q5 for inpatient care.

Another perspective to express equality is CI. The corresponding CI showed that the overall inpatient care utilisation was generally equal or slightly pro-poor throughout the years of the survey (Table 1). Outpatient utilisation was slightly pro-rich prior to 2006, and then become equal, before changing back to slightly pro-rich in 2015. As for dental care visits, it remained pro-rich since 1996 and persisted throughout the recent survey in 2015.

Inpatient utilisation at public providers showed a persistent trend over the years, as demonstrated by the CI (Table 2). The trend exhibited increased utilisation among the poorer population, with a CI range between −0.11 and −0.15. Outpatient utilisation at public providers exhibited similar trends. CI was −0.07 in 1986 and climbed to −0.20 (becoming more pro-poor); outpatient utilisation remained pro-poor until 2015.

Meanwhile, utilisation at private providers showed the opposite tendencies (Table 3). Inpatient care utilisation showed high pro-rich values ranged from 0.31 to 0.52 throughout the years the survey was conducted. Similarly, outpatient utilisation was steadily pro-rich, although not to the extent of inpatient care. CI ranged between 0.13 and 0.26. Nonetheless, the pro-rich trend slightly decreased throughout the years.

## Discussion

The results of the present study showed the inequity in healthcare utilisation between the rich and the poor and the slight changes in trends over time in Malaysia. The survey, started in 1986 and most recently conducted in 2015, revealed that the overall utilisation for outpatient and inpatient visits was close to being equal, based on the CI values. However, inequalities can be seen when the analysis divided the providers to public and private. The pro-poor utilisation (of inpatient and outpatient care) in public providers and pro-rich pattern in the private sector generally persisted over time. Meanwhile, dental care utilisation was pro-rich throughout the survey period.

Findings from this study match the outcomes in neighbouring and other developing countries. Utilisation in low- and middle-income countries has shown disparities between the rich and the poor in both inpatient and outpatient care. In India, the inequity in outpatient and inpatient utilisation was demonstrated across the rural and urban populations in a national survey (17), where pro-rich utilisation was observed across all states. In Thailand, with the adaptation of a universal health insurance, healthcare utilisation in outpatient care grew more concentrated among the poor, whereas inpatient care was highly utilised by the better off. However, two consecutive national surveys in 2001 and 2005 demonstrated that the inequity gap was closing, although the pro-rich and pro-poor utilisation between the two health sectors persisted (18). In contrast, gaps were widening between the rich and the poor from 1998 to 2007 in healthcare utilisation in the Philippines (19). Nonetheless, the lack of recent findings on equity in the Philippines limits the comparison to be drawn with Malaysia. Despite the implementation of a National Health Insurance Program in Philippines and progressing towards UHC, disparities remained in terms of healthcare services utilisation.

In Malaysia, the possible reasons for inequity in healthcare utilisation between the rich and the poor are rooted in the division of services into the public and private sectors. Private providers are most commonly located in urban areas, based on the demands of the affluent local community, thereby increasing the healthcare utilisation of private providers (5). The domination of the rich in the utilisation of inpatient care in the private sector may also be attributed to the sector requiring out-of-pocket payment and the availability of insurance reimbursement to the rich. Meanwhile, the pro-poor utilisation of public providers may be due to the affordability of the services, which are largely funded by the government. The variation in SES resulted in discrepancies in the use of such services. As for the pro-rich utilisation of dental services, the reason may be the geographic location of the dental facilities, high cost of services, and post-operative complications (20). A consequence of such disparities is the poor health outcome and poor health status in the lower SES populations.

This study has several limitations. First, methodological differences between surveys and inconsistencies in the survey questions have been reported (10). Second, the cross-sectional survey design could not directly establish causal relations. Indeed, there are many other determinants, such as acceptability and quality of care, which can affect healthcare utilisation (21). Third, this study did not consider health needs across SES segments; those with higher needs should have higher utilisation. Future work in which the analysis further segregates the sample into urban and rural users, as well as male and female, or uses stepwise age would show more specific patterns of utilisation in Malaysia. The inclusion of health need variables in the analysis could also provide a better view on healthcare utilisation equity.

## Conclusion

The effect of pro-poor utilisation in the public sector was generally cancelled out by the pro-rich utilisation in the private sector, leading to a total health utilisation that is almost equal. Although it is commendable that the public sector exhibits a pro-poor utilisation, as desired by the government, it is also equally important to formulate strategies to allow and attract healthcare utilisation of the private sector, which is currently dominated by the richest (Q5), especially by users in rural and remote areas, to reduce congestion in the public sector and improve equity in the utilisation of healthcare services. The early hypothesis on the shift in healthcare utilisation from private to public sector was not clearly seen. This study found slight increases of recent private utilisation among Q5, which may indicate that only the Q5 population can afford the private sector, especially for inpatient care.

## Figures and Tables

**Table 1:** Distribution of health utilisation to public and private providers by SES quintile, 1986–2015

Survey Year	SES Quintile					Concentration Index (CI)	*P*-value

	Poorest 20%	Q2	Q3	Q4	Richest 20%		
Inpatient^1^							
1986	22	22	10	26	21	−0.01	0.910
1996	23	21	19	20	18	−0.06	0.017
2006	21	21	19	20	18	−0.03	0.021
2011	20	25	16	27	13	−0.06	0.360
2015	24	25	17	22	12	−0.02	0.416
Outpatient^2^							
1986	16	18	20	22	25	0.10	< 0.001
1996	17	20	19	22	21	0.04	< 0.001
2006	20	20	20	21	19	−0.01	0.466
2011	16	22	21	22	18	0.01	0.658
2015	24	26	22	17	11	−0.13	< 0.001
Dental^3^							
1986	N/A	N/A	N/A	N/A	N/A	N/A	
1996	18	20	20	19	23	0.05	< 0.001
2006	19	19	20	21	21	0.03	< 0.001
2011	15	18	19	21	27	0.12	< 0.001
2015	14	19	20	22	25	0.16	< 0.001

Source: NHMS 1986–2015

Notes:

1 Based on inpatient days, with exception of NHMS 2006 which uses inpatient visits

2 Based on outpatient medical visits

3 Based on dental care visits, no data for NHMS 1986

**Table 2:** Distribution of health utilisation to public providers by SES quintile, 1986–2015

Survey Year	SES Quintile					Concentration Index (CI)	*P*-value

	Poorest 20%	Q2	Q3	Q4	Richest 20%		
Inpatient^1^							
1986	24	25	11	28	13	−0.11	0.196
1996	25	22	20	20	14	−0.12	< 0.001
2006	25	23	21	19	12	−0.12	< 0.001
2011	23	28	16	25	8	−0.15	0.061
2015	27	28	19	18	8	−0.18	< 0.001
Outpatient^2^							
1986	22	22	21	21	14	−0.07	< 0.001
1996	29	27	19	15	10	−0.21	< 0.001
2006	29	25	20	15	11	−0.20	< 0.001
2011	23	30	24	15	9	−0.18	< 0.001
2015	25	26	22	16	11	−0.17	< 0.001

Source: NHMS 1986–2015

Notes:

1 Based on inpatient days, with exception of NHMS 2006 which uses inpatient visits

2 Based on outpatient medical visits

**Table 3:** Distribution of health utilisation to private providers by SES quintile, 1986–2015

Survey Year	SES Quintile					Concentration Index (CI)	*P*-value

	Poorest 20%	Q2	Q3	Q4	Richest 20%		
Inpatient^1^							
1986	15	4	2	17	63	0.52	0.080
1996	11	10	14	22	42	0.31	< 0.001
2006	4	10	10	24	52	0.46	< 0.001
2011	4	13	14	34	35	0.35	< 0.001
2015	7	8	11	38	36	0.40	< 0.001
Outpatient^2^							
1986	10	13	18	23	36	0.26	< 0.001
1996	11	16	19	26	28	0.19	< 0.001
2006	14	17	19	26	25	0.13	< 0.001
2011	10	15	19	29	27	0.20	< 0.001
2015	11	21	25	22	21	0.24	0.031

Source: NHMS 1986–2015

Notes:

1 Based on inpatient days, with exception of NHMS 2006 which uses inpatient visits

2 Based on outpatient medical visits
